# The transcriptional signature associated with human motile cilia

**DOI:** 10.1038/s41598-020-66453-4

**Published:** 2020-07-02

**Authors:** Anirudh Patir, Amy M. Fraser, Mark W. Barnett, Lynn McTeir, Joe Rainger, Megan G. Davey, Tom C. Freeman

**Affiliations:** The Roslin Institute, University of Edinburgh, Easter Bush, Midlothian, Scotland EH25 9RG UK

**Keywords:** Cell biology, Computational biology and bioinformatics, Systems biology

## Abstract

Cilia are complex microtubule-based organelles essential to a range of processes associated with embryogenesis and tissue homeostasis. Mutations in components of these organelles or those involved in their assembly may result in a diverse set of diseases collectively known as ciliopathies. Accordingly, many cilia-associated proteins have been described, while those distinguishing cilia subtypes are poorly defined. Here we set out to define genes associated with motile cilia in humans based on their transcriptional signature. To define the signature, we performed network deconvolution of transcriptomics data derived from tissues possessing motile ciliated cell populations. For each tissue, genes coexpressed with the motile cilia-associated transcriptional factor, *FOXJ1*, were identified. The consensus across tissues provided a transcriptional signature of 248 genes. To validate these, we examined the literature, databases (CilDB, CentrosomeDB, CiliaCarta and SysCilia), single cell RNA-Seq data, and the localisation of mRNA and proteins in motile ciliated cells. In the case of six poorly characterised signature genes, we performed new localisation experiments on *ARMC3*, *EFCAB6*, *FAM183A*, *MYCBPAP, RIBC2* and *VWA3A*. In summary, we report a set of motile cilia-associated genes that helps shape our understanding of these complex cellular organelles.

## Introduction

Cilia and flagella are related organelles that facilitate an array of cellular functions. In eukaryotes, the core structural components of cilia includes: the *axoneme*, a microtubular protrusion from the cell surface composed of an array of microtubules; a *centrosomal core*, comprised of a mother (basal body) and daughter centriole^1,2^ anchored to the base of the axoneme, and the centriole-associated distal and sub-distal appendages^3^. Generally, cilia can be subdivided into non-motile primary cilia, in which nine microtubules constitute the axoneme (9 + 0) and motile cilia, characterised by an additional central pair of microtubules (9 + 2)^4–6^. Primary cilia are found on most cell types, where their principal role is as a sensor of the cell’s microenvironment^7^. In contrast, motile cilia are restricted to specific cell populations. Flagellum function as a single large ‘propeller’ and in eukaryotes are found exclusively on spermatocytes where they drive cell motility. Other motile cilia are found in large numbers on the apical surface of certain types of epithelial cells, where their co-ordinated beating displaces the luminal contents over the epithelial surface, e.g. the clearance of mucus in the respiratory tract. Whilst there are a set of core proteins common to all cilia, there are also structural and regulatory elements unique to motile cilia which underpin their distinct functional activity^8,9^.

Motile cilia play a vital role in human development and homeostasis, and there is a growing list of ciliopathies (cilia-related diseases) associated with mutations of ciliary assembly proteins and protein components of these organelles. These include defects in left-right patterning during embryogenesis^10^, infertility^11^, asthma^12^ and hydrocephalus^13^. Perhaps the most notable and well-characterised ciliopathy is primary ciliary dyskinesia, an autosomal recessive disorder which has an estimated prevalence of 1 in 10,000^5,14^. Causative mutations leading to primary ciliary dyskinesia include those in genes encoding the motile ciliary components of radial spokes (*RSPH1*, *RSPH9* and *RSPH4A*)^15,16^, dynein arms, specifically the outer dynein arm (*DNAI1*, *DNAI2* and *DNAH11*)^17–19^, proteins involved in their assembly (*CCDC103, LRRC6* and *ZMYND10*)^20–22^ and the key transcriptional regulator of motile ciliogenesis *FOXJ1*^23^. Patients carrying mutations in these genes are often treated for respiratory symptoms, including chronic respiratory infections, due to their inefficient clearance of mucus from the lungs^24,25^.

There have already been considerable efforts made to characterise the molecular components of cilia. FOXJ1 and the RFX family of genes have been identified as the key transcription factors which regulate motile ciliogenesis, which in turn, depending on species, have shown to be regulated by the Wnt, Hedgehog and Notch signalling pathways^9^. Moreover, in conjunction with other transcriptional regulators, such as HNF1B and SOX5, further ciliary diversity is introduced for mechanosensory renal cilia and bronchiolar cilia, respectively^26,27^. Proteomic profiling studies have sought to define the components of motile cilia by dysregulating such transcriptional regulators and analysing the proteome of isolated cilia preparations using mass spectrometry^28–32^. Each of these studies has produced a list of cilia-associated proteins and accordingly a number of databases have been established. The most relevant to the study of human cilia include: *CentrosomeDB*, a set of human (and *Drosophila*) genes encoding proteins that are localized in the centrosome, either as centrosome constituents or as centrosome visitors^33^; *CilDB* a database dedicated to proteins involved in centrioles, centrosomes, basal bodies, cilia and flagella in eukaryotes^34^; *SysCilia* a curated list of cilia genes many of which are associated with disease^35^; and *CiliaCarta* which employs a naive Bayesian classifier to predict cilia candidate genes across a diverse set of datasets^36^. These resources list between 303 and 3,376 genes and have greatly broadened our understanding of the complexity of cilia while attempting to define the role of these genes in the context of development, ciliogenesis and ciliopathies.

Here we have sought to provide a consensus human motile cilia gene signature conserved across known motile cilia containing tissues and compare it with the relevant databases. We have used a network deconvolution approach to define gene coexpression clusters containing the transcriptional regulator *FOXJ1*^37^ using transcriptomics data from the Genotype-Tissue Expression (GTEx)^38^ project for human tissues known to possess motile ciliated cells. In support of these analyses, we have also examined various lines of evidence in order to validate the set of genes identified. These include a comparison with cilia and centrosomal databases mentioned above, studies of their expression profile across motile and primary cilia containing cells and tissues, and a number of new expression studies examining several poorly characterised genes identified by this work, namely *ARMC3*, *EFCAB6*, *FAM183A*, *MYCBPAP*, *RIBC2* and *VWA3A*. Overall the study proposes a set of motile cilia cilia-associated associated genes that are tightly coexpressed across tissues, including certain but not all cilia-associated centrosomal genes previously identified. The signature genes have been summarised graphically based on their function and/or known localization.

## Results

### Derivation of the human motile cilia signature

**Figure 1:**
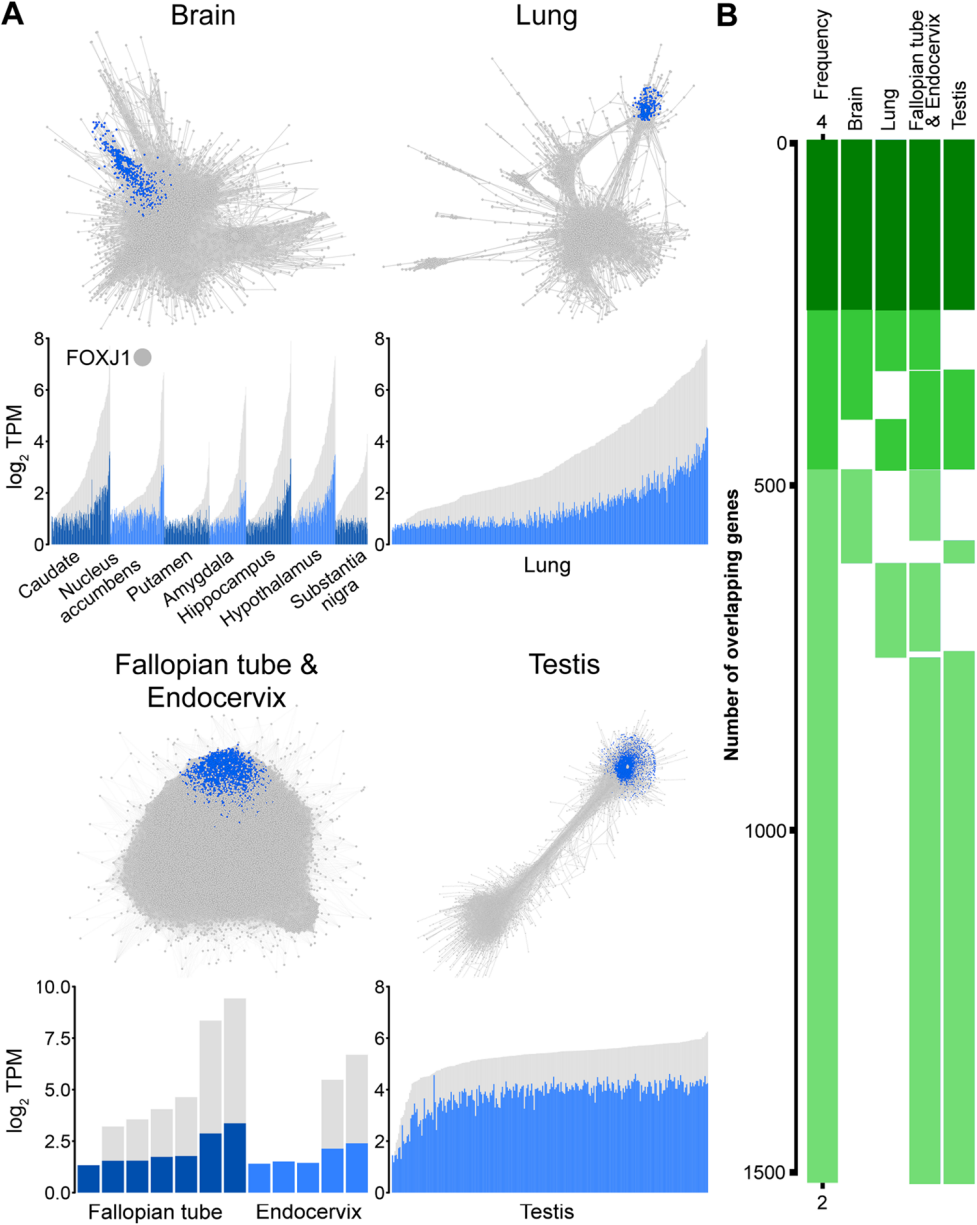
Derivation of the human motile cilia transcriptomic signature. **(A)** Gene coexpression networks for each of the four selected tissue types generated from RNA-Seq data from the GTEx project. Each node represents a gene, with genes sharing similar expression profiles being highly connected to one another; genes that co-clustered with *FOXJ1* have been highlighted in blue in the network. The corresponding expression profile is shown for *FOXJ1*-containing gene clusters (blue) with the average expression of its genes and individually shown for *FOXJ1* (grey). Here, each column represents individual samples of the respective tissue. **(B)** 1,517 genes were found to be present in more than one of the four *FOXJ1*-containing gene clusters. Genes present in all four tissue clusters are depicted in dark green and represent the final 248 tissue-derived motile cilia gene signature.

**Figure 2:**
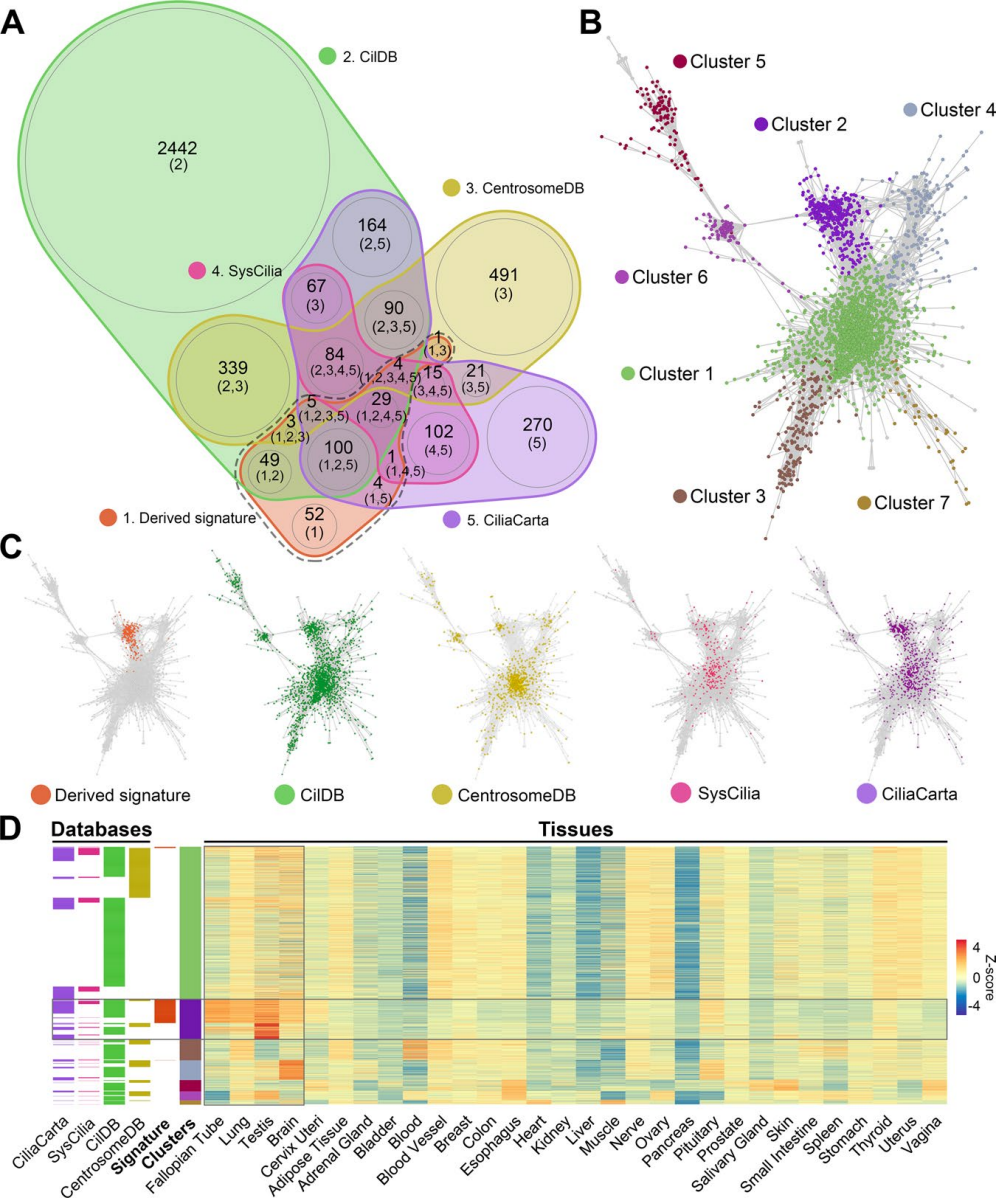
Comparison of signature and database genes and their expression across tissues. **(A)** Venn diagram of overlapping genes between the derived signature and databases using nVenn v0.2.1 (https://cran.r-project.org/web/packages/nVennR/index.html)^77^. Here the overlap between gene lists can be observed through the overlap of their respective segments and the numbers shown in brackets, are indicative of the gene list number. The derived signature is encircled by a dashed line. **(B)** Gene coexpression network constructed in Graphia v1 (https://kajeka.com/graphia/) for all these genes as defined by their expression in the context of GTEx tissues. Nodes are coloured by cluster analysis of this network. **(C)** Genes from the signature and included in each of the databases have been highlighted individually on the network. **(D)** Heatmap of the signature and database genes across GTEx tissues ordered based on the gene clustering from **(B**).

The GTEx RNA-Seq (v7) dataset is the largest transcriptomics data resource for non-pathological human tissues currently available and was used here to derive a human motile cilia gene signature. Data derived from tissues known to contain cell populations possessing motile cilia, i.e. ependymal cells in brain regions likely to adjoin the cerebrospinal fluid-filled ventricular space (n = 863), bronchial epithelia of the lung (n = 427), spermatocytes in testis (n = 259) and tubal epithelial cells in fallopian tube/endocervix (n = 12) were downloaded; in total this represented tissue RNA-Seq data from 1,561 samples derived from 566 donors (Fig. 1). To identify genes associated with motile cilia, we examined coexpressing genes, i.e. genes which have a similar expression profile across samples from the same tissue. This similarity was measured using the Pearson correlation coefficient and highly correlated genes were used to construct a gene correlation network (GCN). The network comprises of nodes representing genes, and those correlated beyond the selected threshold are connected within the network. Subsequently, the network was subjected to network cluster analysis to define groups of coexpressed genes, and in the case of each tissue GCN, those that clustered with *FOXJ1* (Table S1). *FOXJ1* associated clusters ranged in size from 597 to 6,126 genes. Such variation in the size of the motile cilia cluster across tissues is likely explained by a varying number of samples and the different tissue biology, e.g. the expression landscape of the testis is dominated by transcriptional signal associated with spermatogenesis, making the flagellum-specific gene module difficult to separate from other sperm-associated gene clusters^39^. 1,517 genes from the different tissue derived signatures overlapped with one another (Fig. 2B), and for the final signature we considered only the 248 genes present in all tissue-derived gene lists. However, we acknowledge that the extended list, i.e. 479 genes found in three of the four motile cilia tissue clusters contains many additional validated cilia genes and therefore is likely to contain many other novel motile cilia-associated genes (Table S1).

### Comparison with database genes and their expression profiles across cells/tissue

Enrichment analysis of the 248 gene signature was conducted for GO terms, pathways, gene families, transcription factor binding sites, and human phenotypes (Table S2). Enriched gene families included components of the ‘dynein regulatory complex’ (*q* value = 2.2 × 10^−15^), ‘axonemal, dyneins’, (*q* value = 3.2 × 10^−13^) and ‘tektins’ (*q* value = 1.4 × 10^−7^), with the corresponding enrichment of biological processes such as ‘cilium movement’ (*q* value = 3.4 × 10^−52^), ‘cilium organization’ (*q* value = 5.1 × 10^−40^) and ‘cilium-dependent cell motility’ (*q* value = 2.7 × 10^−26^). Additionally, binding sites for RFX1 and MIF were also found to be enriched for these genes (*q* value < 10^−7^). Human disease phenotypes associated with disorders of motile cilia included ‘abnormal respiratory motile cilium morphology’ (*q* value = 1.2 × 10^−24^), ‘situs inversus totalis’ (*q* value = 3.4 × 10^−21^), ‘bronchiectasis’ (*q* value = 1.5 × 10^−13^), and ‘male infertility’ (*q* value = 3.7 × 10^−13^). Signature genes associated with human ciliopathies were identified from OMIM, a knowledgebase of human genetic disorders^40^. The database listed 33 of the signature genes linked to human ciliopathies, majority being primary ciliary dyskinesia (Table S2). To further investigate the ciliary/centrosomal association of the signature genes we first conducted a literature search on all signature genes. Of the genes identified, the literature supported 133 (54%) as having direct experimental evidence supporting their spatial localization or functional association with cilia (Table S1). A further 87 (35%) genes were found associated with cilia through coexpression analysis but without any direct evidence of their localization within ciliary structures. For 28 genes (11%) no prior association with cilia could be identified.

We then sought to examine the signature’s overlap with public databases of cilia/centrosome proteins. Including the signature reported here, a total of 4,333 genes have been implicated previously with cilia and/or centrosomes. These include the CentrosomeDB^33^, CilDB^34^, CiliaCarta^36^ and SysCilia (gold standard)^35^ (Fig. 2A and Table S3). There were only four genes which were common to all the databases and the derived signature (*DNAAF1*, *FOXJ1*, *KIF24*, and *MAK*). In support of our literature search, the majority of the signature genes (196 genes) overlapped with genes listed in CilDB, including well known motile cilia genes such as members of the dynein regulatory complex (*DRC1*, *TCTE1* and *IQCD*), axonemal dynein (*DNAH2*, *DNALI1* and *DNAI1*) and tektin gene family (*TEKT1*, *TEKT2*, and *TEKT4*), whilst also included genes with poor evidence supporting an association with cilia, e.g. *MYCBPAP*, *ARMC3* and *EFCAB6*. Relative to the databases, 52 genes were found to be unique to the current study and included genes not associated with human motile cilia previously, e.g. *FAM183A* and *VWA3A*. By contrast, 84 genes recorded by all database resources were absent from the derived signature. Upon inspection, these largely represented genes associated with the cell cycle^41^ and ciliary assembly and maintenance, e.g. members of the centrin family (*CETN1*, *CETN2* and *CETN3*), BBSome complex members (*BBS1*, *BBS4*, *BBS5* and *BBS7*) and IFT genes (*IFT20*, *IFT74* and *IFT81*)^42^.

**Figure 3:**
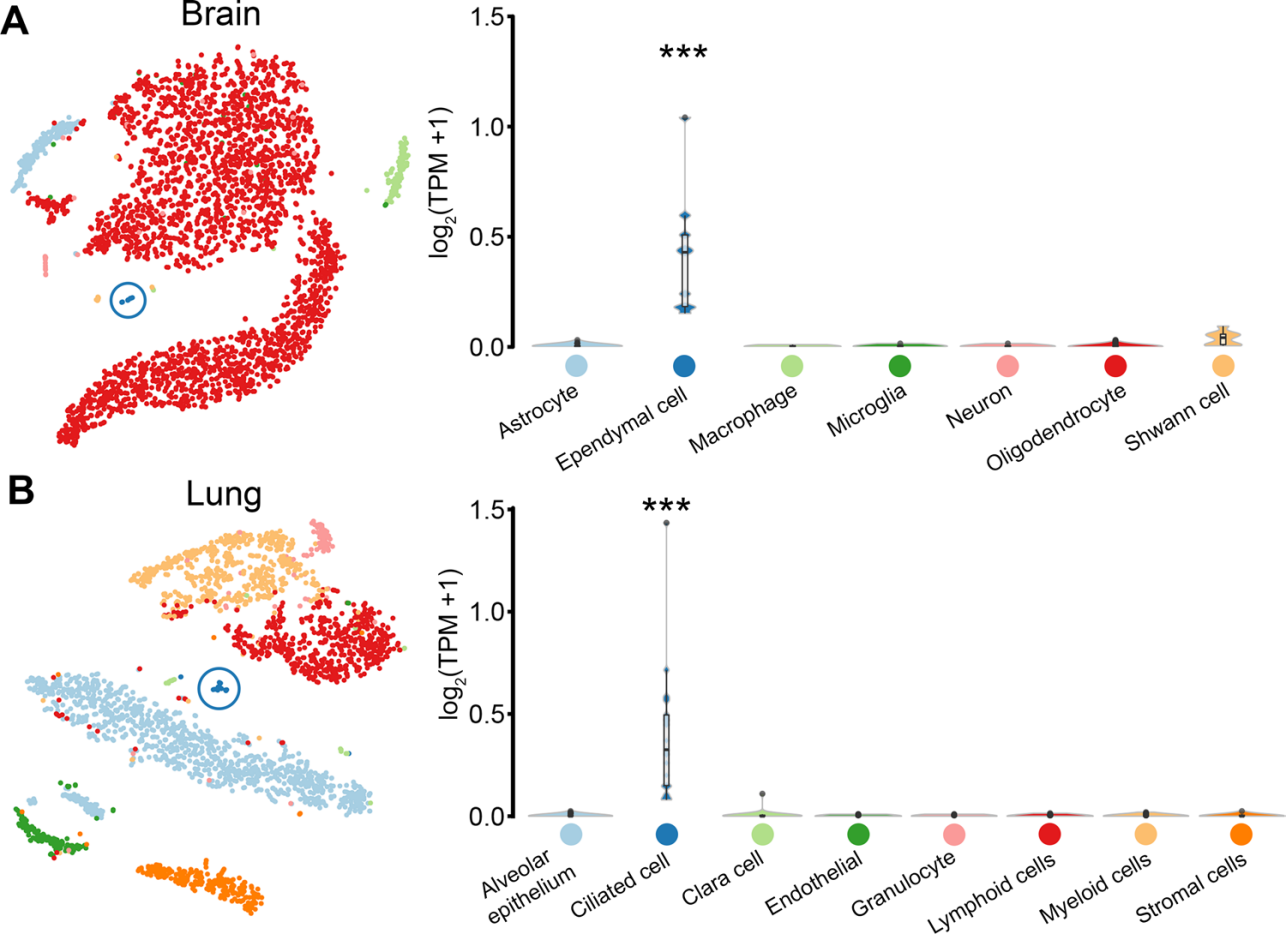
Expression of signature genes in cells from the brain and lung. Average expression of signature genes across cells of the **(A)** brain and **(B)** lung taken from the mouse cell atlas dataset^71^.

As a further analysis, we examined the global expression patterns of all signature genes and those recorded in databases for their expression across all 51 tissue types in the GTEx resource. GCN analysis was again used to visualise and explore the expression profile of signature and database genes across human tissues (Fig. 2B–D). Cluster analysis was used to broadly group genes together based on their underlying expression pattern (Fig. 2B). Highlighting the genes from each database showed them in each case to be distributed across the network. In contrast to the distribution of signature genes which were far more localized. This is indicative of their tight coexpression across all tissues (Fig. 2C), attributed by their relatively high expression in tissues known to have motile ciliated cells (Fig. 2D). Conversely, genes for each of the databases were scattered throughout the graph suggesting that they had very different expression profiles, ranging from a broad expression across all tissues as represented by cluster 1, to being highly expressed in certain tissues such as blood (cluster 3) or brain (cluster 4). Additionally, cluster 3 included many immune genes, e.g. associated with MHC class 1 and 2, TLR receptors and TNF family of genes (Table S3). As a more direct comparator, the expression of signature genes was examined in single cell RNA-Seq data derived from the mouse brain and lung (Fig. 3). Here motile ciliated ependymal and bronchial cells, respectively, showed a significantly higher average expression of signature genes (*q* value < 0.001) when compared to other cell types, again supporting their specific association with motile cilia possessing cells.

### The localisation of candidate proteins in motile ciliated tissue

**Figure 4:**
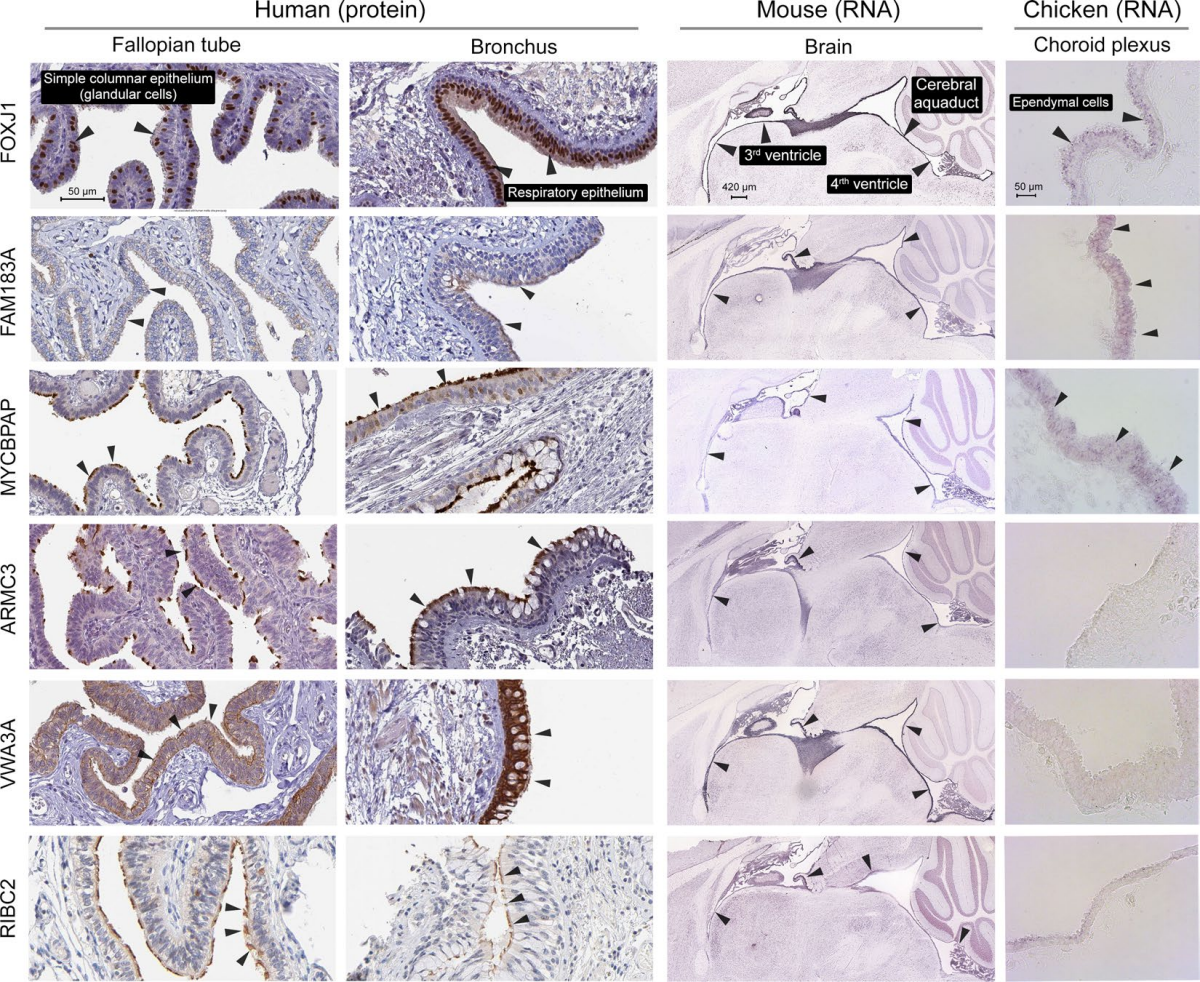
IHC and ISH staining from signature genes across species and tissues. IHC and ISH staining of tissue sections from the HPA and Allen brain atlas of the mouse brain for encoded proteins and transcribed RNA of signature genes, respectively. The final column consists of new ISH staining performed in choroid plexus sections from chicken embryos (stage 35).

In order to provide an additional level of validation for the 248 signature genes, we examined the immunohistochemistry (IHC) data in the Human Protein Atlas (HPA) resource^43^ in tissues containing motile ciliated cells (Fig. 4, Table S1). Based on our own criteria (independent of the confidence scores set by HPA), genes were placed into three groups: high confidence genes (n = 119, 48%) were those where positive staining for the cilia/centrosome was observed in at least one tissue with no staining of other structures. Medium confidence (n = 50, 20%) was assigned to genes where the protein was positively stained for in cilia/centrosomes, but the data also showed staining of other structures. Finally, for 79 (32%) genes no data was available or no apparent staining was observed on the sections, and they were designated as being unsupported by this approach. In no cases did we observe any evidence of the specific staining of non-ciliated cells.

### Experimental validation of uncharacterised cilia-associated genes

After collating the results of the above analyses, six genes with little evidence in the literature of an association with cilia were selected for further investigation. This included five high confidence genes based on our assessment of the HPA IHC data: *ARMC3, FAM183A, MYCBPAP*, *RIBC2* and *VWA3A*, and *EFCAB6* which had no HPA data associated with it. For these genes, we examined localised gene expression through RNA *in situ* hybridisation (ISH) data from the Allen mouse brain atlas^44^. Furthermore, we performed ISH analyses on sections of the choroid plexus from chicken embryos (stage 35) looking for staining in ependymal cells (Fig. 4 and S1) which have motile cilia^45^. In all cases, positive staining for motile ciliated cells was observed in the mouse brain, however, ISH staining of ependymal cells lining the choroid plexus in the chickens was only observed in the cases of *EFCAB6, FAM183A* and *MYCBPAP*.

**Figure 5:**
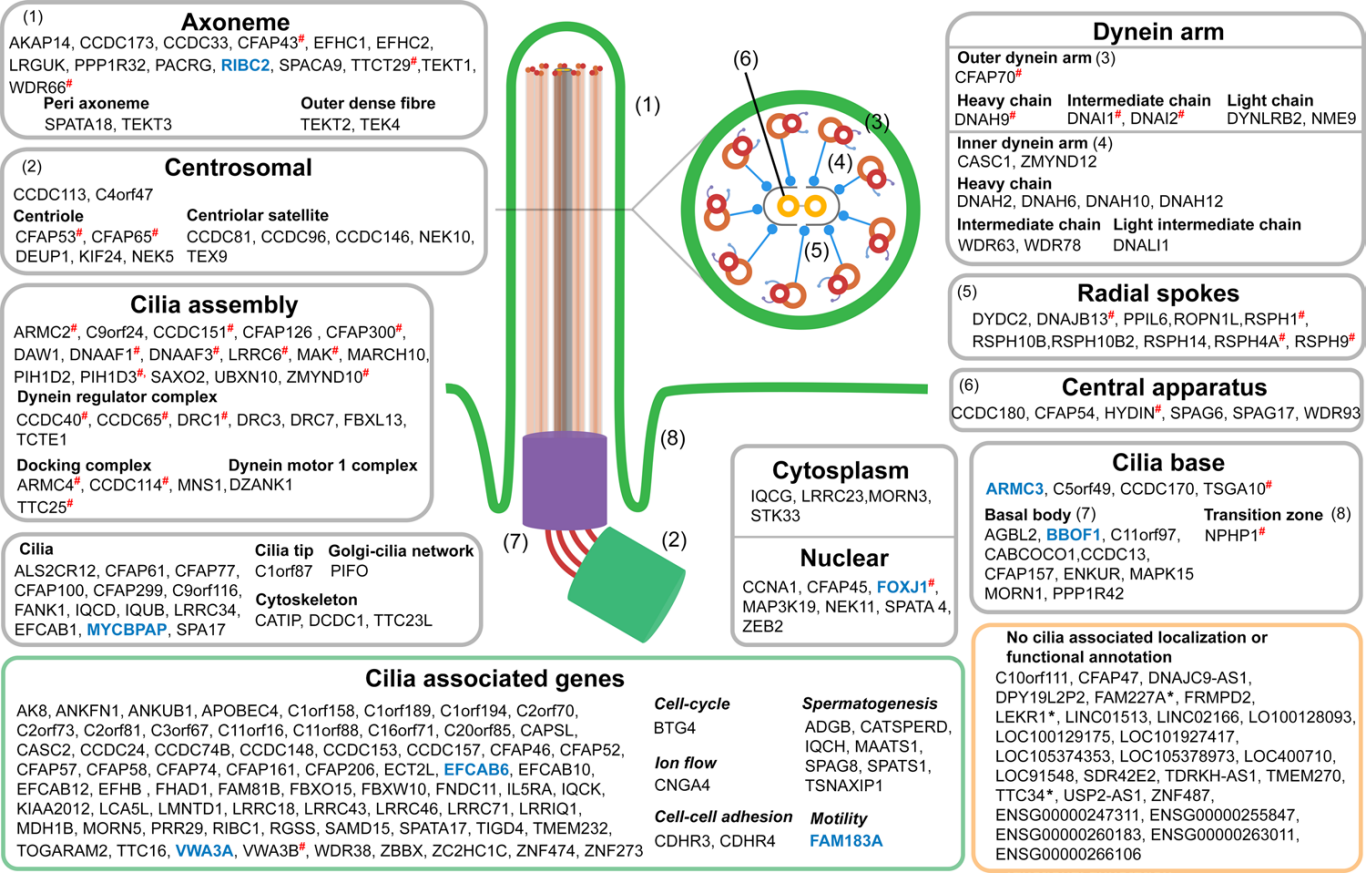
Annotation of derived signature genes. Functional annotation of signature genes in the context of cilia based on previous literature, giving priority for their spatial localization. Genes with evidence for their localization within the context of cilia are grouped in grey boxes. Genes associated with cilia, but lacking any localization evidence are grouped in the green box. Those without any previous evidence of an association with cilia are grouped in the yellow box, with those being scored high or medium confidence genes based on our analyses being marked with an asterisk. Additionally, genes examined experimentally in this study are highlighted in blue and those linked to human ciliopathies based on OMIM^40^ are marked with a red hash sign.

As a graphical summary of this work, we sought to categorize all motile cilia signature genes based on their known association with cilia (Fig. 5, Table S1). The figure broadly categorized genes into different levels of confidence based on literature mining, including those with a known localization (grey box), cilia-association but no localization (green box) and those with no association at all (yellow box). Genes examined experimentally here are highlighted in blue and those associated with human ciliopathies have red hash symbols.

## Discussion

Motile cilia are a distinct class of cilia. They are characterized by a 9 + 2 configuration of central microtubules, radial spokes and dynein arms, along with specialized proteins involved in their assembly. Together, the microtubules and associated molecular motors allow cilia to beat in an ATP-dependent manner^46^. They are vital in cell motility, embryonic patterning, fertilization and the movement of luminal contents over epithelial surfaces. Mutations in the components of these organelles result in a wide range of disorders termed as ciliopathies. Studies in humans and model organisms have identified thousands of proteins as potentially being involved in cilia biogenesis, maintenance and assembly, and the results of these studies are presented in a number of databases, i.e. CentrosomeDB^33^, CilDB^34^, CiliaCarta^36^ and SysCilia (gold standard)^35^. One of the challenges in identifying candidate genes or proteins specific to motile cilia is the fact that many components are involved in other cellular processes or structures. For example, centrosomal replication is associated with cell division, during which many components are upregulated^41^, and primary cilia, which contain many of the same proteins, are present in most cell types.

Here we have attempted to harness the power of GCN’s and employ the principle of ‘guilt-by-association’ to identify genes specifically associated with motile cilia. This is based on the fact that genes specifically associated with a given cell type or biological process frequently vary in expression with their relative abundance or activity within a sample and consequently across a large sample set the expression of these genes is tightly correlated. This approach has been used previously to identify genes associated with specific cell populations and processes, from tissue and cell-level transcriptomics data^47–50^. Here we analysed the brain, lung and the female reproductive tract, all of which contain populations of multi-ciliated cells which function to move luminal contents (cerebrospinal fluid, mucus) over the epithelial surface. In addition, we examined the testis, where cilia proteins are associated with the flagellum of sperm, a fundamentally different type of motile cilia but comprised of many of the same molecular components. In the current study, we first identified genes from each of the selected tissues which co-clustered with *FOXJ1*, a key transcriptional regulator of the motile ciliogenic program^37,51,52^. In the case of the brain and lung, a clear transcriptional module associated with multiciliated epithelial cells was defined due to the marked variation in the abundance of these cell populations across the samples. For the female reproductive tract and testis, however, such modules were harder to define accurately, as there were either only a few samples available and with flagellum-related genes being strongly associated with genes involved in spermatogenesis^39^, respectively. To circumvent these limitations and filter out any cell type-specific genes, we compared the gene clusters from each tissue to arrive at a consensus signature of 248 genes. It should be noted, however, that the list of genes associated with three of the tissue clusters (an additional 231 genes) also contained many other known cilia proteins and therefore by inference genes encoding other uncharacterised cilia components (Table S1).

Validation of the gene signature included enrichment analyses, annotation based on a literature review and cilia-associated databases, and exploration of other resources describing the cellular expression of genes and proteins, confirmed the majority to be known components of motile cilia or associated regulatory systems (summarised in Fig. 5). The binding site for the transcriptional factor RFX1, a member of the RFX gene family^53^, was enriched. This gene has shown to be involved in development, based on a mouse knockout model and regulates the basal body-associated protein ALMS1, defects in which cause Alström syndrome ciliopathy^54,55^. Furthermore, another member, RFX2 has been proposed to work in conjunction with FOXJ1 to regulate cilia gene expression, including RIBC2 also identified and examined in this study^56,57^. However, of the RFX family only RFX3 was found in three of the tissue-derived motile cilia signatures, making it a likely candidate in regulating motile-ciliogenesis, an observation supported by a recent study^58^. In addition, the transcriptional binding site for MIF was also enriched in signature gene promoters. MIF is known to affect cell motility through the regulation of microtubule formation^59,60^.

The signature genes were also cross-referenced with the four cilia/centrosome gene databases; CentrosomeDB^33^, CilDB^34^, CiliaCarta^36^ and SysCilia (gold standard)^35^ databases. The majority (79%) of genes in the signature were corroborated by one or more of the databases. Notably, many well-known primary cilia genes involved in ciliary assembly and signalling and listed by the databases were absent from the signature. This included members of the BBSome complex, IFT chain^61,62^ and many associated with the centrosome. As known components of primary cilia, these genes are regulated through the stages of cell cycle and are ubiquitously expressed across cell types, and would therefore be expected to have a different expression profile in the tissues examined relative to the genes coexpressing with *FOXJ1*. To explore the expression of signature and database genes across tissues, GCN analysis was used for all the 51 tissues from the GTEx project. Interestingly, although the signature was derived from separate analyses of individual tissues, in general their coexpression was highly conserved across the 51 tissue types, being highly expressed in motile ciliated tissues relative to others. Interestingly, these also included a number of centrosomal genes indicative of a specialised centrosomal system for motile cilia assembly and function. Genes listed by the various databases coexpressing with those of the signature included known motile cilia components like dyneins (*DNAH3*, *DNAH7*, and *DNAH8*) and members of the α-tubulin gene family (*TUBA3D*, *TUBA3E*, and *TUBA3C*)^63,64^. In contrast, analysis of databases showed genes within a given database to be distributed across the GCN, and exhibit little evidence of co-expression, suggestive of representing different biology across tissues. Closer inspection showed some to be immune-related genes, e.g. TLR and MHC genes, and their presence in the databases is likely an artefact of the approaches used to define them^65^. As a direct validation of their specificity of expression, single-cell transcriptomics data derived from the mouse brain and lung showed the signature genes to be highly and specifically expressed in ependymal and ciliated epithelial cells, respectively, of these tissues. Hence, the signature as a whole could help in identifying motile cilia biology in bulk and single cell transcriptomics data due to its conserved nature across several cells and tissues. Furthermore, in representing a homeostatic motile cilia, the signature would be a reference to motile cilia in disease models.

The HPA resource was used to further verify the validity of signature genes based on IHC analysis. Genes for which there was data were ranked as being of either high or medium confidence based on their expression pattern matching that expected for proteins associated with motile cilia. Our analysis of the HPA data showed it to validate the majority of signature genes; 48% were scored as high confidence genes and 20% as medium confidence, based on the criteria outlined in the methods. Nothing could be concluded for the 32% genes for which no data was available or the data was of very poor quality. Remarkably, none of the protein localization data directly contradicted an association of any gene with motile cilia. We then set out to further investigate six genes, *EFCAB6* having no HPA data and five high confidence genes based on their HPA IHC results but with poor evidence of their association with cilia in humans based on literature: *ARMC3*, *FAM183A*, *MYCBPAP, RIBC2* and *VWA3A*. ISH experiments performed on the chicken choroid plexus, showed *FAM183A, EFCAB6 and MYCBPAP* to be expressed by motile ciliated cells lining this tissue, although this was not apparent for *RIBC2*, *VWA3A* and *ARMC3*. Apart from being a possible false negative, this discrepancy could be indicative of the diversity of ciliary components in eukaryotes^66^. A recent study using evolutionary proteomics has predicted *MYCBPAP* to be associated with cilia, and ISH of the *FAM183A* orthologue has shown to positively stain motile ciliated tissue in *Xenopus laevis*^67,68^. In support of our observations, ISH data from the Allen brain atlas for the selected genes showed positive staining of ciliated cells lining the ventricles of the mouse brain. Finally, we have summarized our findings graphically based on database and literature mining of the known associations of signature genes with cilia structures and function, as well as human ciliopathies. Clearly, many of the known components of the motile cilia machinery have been identified by this study, and many others have evidence supporting their association but not with specific components of the organelle. The curation of the list clearly highlights the many potentially novel cilia genes/proteins identified by this work and those which can be further examined.

In summary, we have used coexpression analyses to identify a set of 248 genes highly associated with the presence of motile ciliated cells within human tissue. Significant efforts were then made to validate the genes identified based on further coexpression analyses, extensive searches of the literature, online resources of information on the cellular and tissue expression data for genes and proteins, as well as public databases of cilia related genes across different species. Along with a graphical description of signature genes within cilia, the signature highlights similar genes from cilia and centrosome databases, helping in the categorization of known cilia genes. In the case of a number of poorly described genes we identified i.e. *ARMC3*, *EFCAB6, FAM183A, MYCBPAP*, *RIBC2* and *VWA3A,* we have been able to provide new evidence supporting their association with motile cilia. Such analyses serve to extend and refine the list of genes/proteins specifically associated with motile cilia, allowing more targeted analyses of their localisation and functional role within these complex and important organelles.

## Material and methods

### Data pre-processing, signature derivation

Pre-normalized RNA-Seq data from the GTEx project^38^ was downloaded (version 7) and log-transformed. Data for tissues known to possess motile ciliated cells were sub-sampled. These included samples taken from seven regions of the brain (n = 863), lung (n = 427), testis (n = 259), fallopian tube (n = 7) and endocervix (n = 5). Due to the small number of samples of fallopian tube and endocervix, data from these tissues were combined. As such, the relative content of motile cilia containing cells varied considerably across samples, with the expression of genes specifically associated with these structures varying accordingly. Motile cilia-associated genes were identified for each individual tissue by GCN analysis. In order to generate a GCN, a gene-to-gene Pearson correlation matrix was calculated between all genes using the network analysis software, Graphia v1 (Kajeka Ltd., Edinburgh, UK, https://kajeka.com/graphia/). A threshold of *r* ≥ 0.8 was then applied such that only genes correlated to others above this threshold were connected by an edge. In each case, a structured GCN was generated with modules of coexpressed genes forming highly connected cliques within the network. These were defined as clusters using the Markov clustering algorithm (MCL)^69^, using an inflation value MCLi = 2.2 (which defines the granularity of clustering). Putative motile cilia-associated genes were defined as those present in the same cluster as *FOXJ1*. Accordingly, four gene clusters were obtained, one for each tissue type. This approach has been adopted previously to identify co-regulated genes with a related function or association with a given cell type^48–50^. From the four tissue-derived signatures, those genes common to all four signatures were considered for the final human motile cilia signature. Evidence for an association with cilia was explored through literature mining and enrichment analysis, conducted using ToppGene^70^. Disease association for genes was found through the OMIM database^40^.

### Functional annotation of motile cilia signature genes and comparison with databases

Evidence for an association of the 248 motile cilia signature genes with cilia was explored through literature mining and enrichment analysis, conducted using ToppGene^70^. Signature genes were then compared to genes listed in the databases of cilia and centrosomal components, i.e. CentrosomeDB^33^, CilDB^34^, CiliaCarta^36^ and SysCilia (gold standard)^35^ were collated based on their Ensembl gene IDs and compared to the signature list derived here (Table S3). The expression profile of this combined list was examined across 51 tissues (excluding samples derived from pooled cells) of the GTEx dataset (n = 11,215, donors = 713). A GCN was then generated using these genes only, again using a correlation threshold of *r* ≥ 0.8 and the resultant graph was clustered using a low inflation value (MCLi = 1.2) so as to provide a coarse grain segmentation of the graph comprising of 17 clusters.

To explore the expression of the signature genes at a cellular level, single cell transcriptomics data from the mouse brain and lung were taken from the Mouse Cell Atlas^71^ and analysed. These tissues were selected as they include populations of motile ciliated cells. The batch corrected expression matrices based on unique molecular identifiers were downloaded from the mouse cell atlas database (https://figshare.com/articles/MCA_DGE_Data/5435866). This included batch 1 of the brain (n = 3285 cells) and lung (n = 2501 cells) cell data. Additionally, for the latter, 11 cells annotated as “dividing cells” were excluded, as it was unclear which cell types these referred to. Corresponding mouse orthologues for signature genes were identified based on their Ensembl gene ID using BioMart^72^. The average expression of signature genes was then tested for significance in the ciliated cell populations (ependymal cells of the brain and ciliated epithelial cells of the lung), versus all other cell types as defined in the mouse cell atlas. The non-parametric Wilcoxon signed-rank test was adopted for these comparisons.

### Immunohistochemistry and RNA *in situ* hybridisation

The tissue distribution of mRNA and proteins for all signature genes were investigated using publicly available resources. IHC staining of human tissue sections from the bronchus and fallopian tube were examined in the HPA^43^. In both tissues, positive staining of the ciliated epithelial cells lining the tissue was considered as validatory evidence.

The expression of a number of novel genes were further examined in the choroid plexus of chicken embryos (stage 35, day 9) by ISH. Chicken embryos were obtained from wild type Isa Brown fertile chicken eggs and were incubated for 9 days and sacrificed as per approval by The Roslin Institute, University of Edinburgh under supervision and approval of a Named Animal Care Welfare Officer in accordance with the regulations prescribed for animals under the Animals (Scientific Procedures) Act 1986, UK. Although not subject to this act, fertilised eggs were monitored throughout incubation to ensure high welfare standards. Researchers involved in this project have the necessary UK Home Office Project and Personal licenses for undertaking experimental protocols involving the use of animals in this project. For selected genes, clones which covered the majority of exons near the centre of the gene were preferentially selected (*ARMC3*: ChEST208k22, *EFCAB6*: ChEST912jB, *FAM183A*: ChEST261m5, *FOXJ1*, *MYCBPAP*: ChEST864g6, and *RIBC2*: ChEST770c15) using the UCSC Genome Browser^73^ and where available obtained (Source BioSciences, UK)^74^. Fertilised chicken eggs were incubated for nine days at which point the embryos were sacrificed, the choroid plexus dissected and tissues fixed overnight in 4% paraformaldehyde (PFA) at 4 °C. Samples were then rinsed in PBS and equilibrated overnight in 15% sucrose/PBS before embedding in sucrose-gelatin (15%:7.5%) and snap frozen in isopentane at −70 °C. Cryostat sections (10 µm) were cut and stored overnight at −20 °C. Sections were then rinsed in PBS and fixed overnight in 4% PFA. After successive rinses with PBS, the tissue was permeabilized by incubation in proteinase-K (20 ng/ml K-03115836001 Roche) for 10 min at room temperature. Sections were treated consecutively with 4% PFA, acetic anhydride solution (0.25% acetic anhydride and 1.3% triethanolamine) with intermittent washing. Finally, 5 nM probe in hybridisation buffer (50% formamide, 5xSSC pH 4.5, 0.05 µg/ml yeast RNA, 0.05 µg/ml heparin, and 1% SDS) was applied to the slides. Following an overnight hybridization with probes at 65 °C, sections went through a series of post-hybridization washes and then maleic acid buffer-tween (0.15 M NaCl, 0.1 M maleic acid, 0.18 M NaOH and 0.02% tween). After blocking (20% heat-inactivated FBS/KTBT) for 1 h, sections were incubated overnight with 1:1000 anti-digoxigenin-alkaline phosphate (11093274910 Roche, 1:1000) at 4 °C. Following a final series of washing with maleic acid buffer-tween, sections were incubated with staining solution (3.5 µl/ml nitro blue tetrazolium, N-6876 Sigma; 3.5 µl/ml BCIP, B-8503). After staining for 1–2 h (depending on the probe), the reaction was stopped by several rinses in PBS. ISH staining of brain sections for these genes was also examined in data from the mouse Allen brain atlas^75^, where the choroid plexus and ventricular system were present (which is lined with motile ciliated ependymal cells).

## Supplementary information

Supplementary Information.

Supplementary Information2.

Supplementary Information3.

Supplementary Information4.

## Data Availability

The tissue and single cell RNA-Seq data that support the findings of this study are available in the Genotype-Tissue Expression portal^38^ (doi: 10.1038/ng.2653., https://gtexportal.org/home/) and from the Mouse Cell Atlas^71^ figshare repostiroy (doi: 10.1016/j.cell.2018.02.001, https://figshare.com/articles/MCA_DGE_Data/5435866) respectively. The cilia associated gene lists used in this study were publically available and taken from the CilDB^34^ website (http://cildb.cgm.cnrs-gif.fr/), CentrosomeDB^33^ website (doi: 10.1093/nar/gkt1126, http://centrosome.cnb.csic.es/), CiliaCarta article^36^ (Table S3, doi: 10.1371/journal.pone.0216705), and Syscilia^35^ website (doi: 10.1186/2046-2530-2-7, http://www.syscilia.org/goldstandard.shtml). Immuohistochemstiry of human tissues that support the findings of this study were publically available in the Human Protein Atlas^76^ website (doi: 10.1126/science.1260419., https://www.proteinatlas.org/). *In situ* hybridyzation images of the mouse brain were publically available in the Allen Brain Atlas website for mouse^75^ (doi: 10.1038/nature05453, https://mouse.brain-map.org/). *In situ* hybrydization experiments conducted on the chicken choroid plexus have been made available within the article and are also available from the corresponding author upon reasonable request.
