# Supplementary figures and images for: The transcriptional signature associated with human motile cilia
Source: Sci Rep. 2020 Jul 2;10:10814. doi: 10.1038/s41598-020-66453-4 (PMC7331728; doi:10.1038/s41598-020-66453-4)

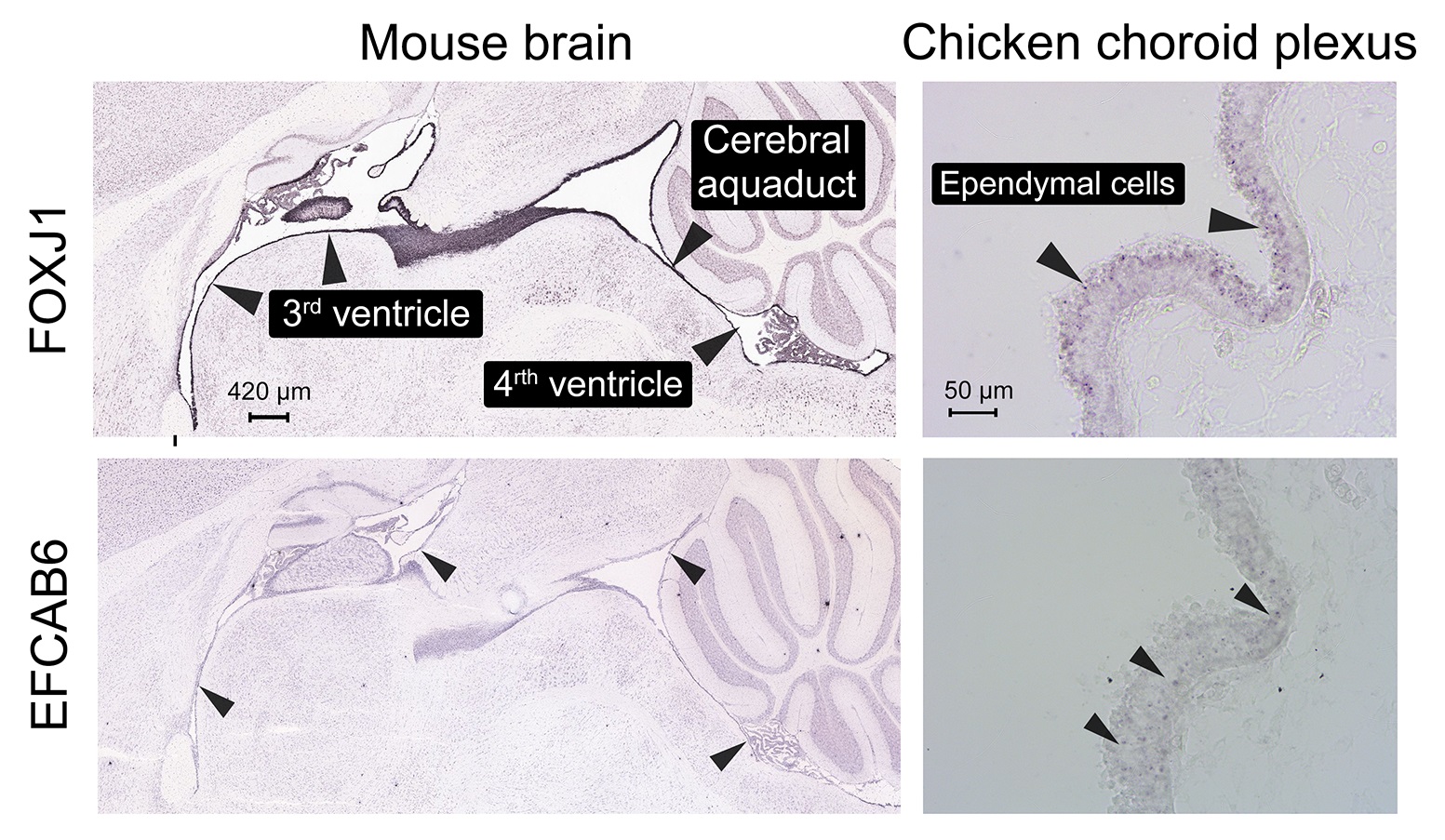

Supplement: Supplementary file 1 — Supplementary Information. [file 41598_2020_66453_MOESM1_ESM.jpg]
